# A Prospective Study Comparing the Functional Outcome of Bipolar Hemiarthroplasty Versus Total Hip Replacement in Elderly Patients With Fracture of the Neck of Femur

**DOI:** 10.7759/cureus.29529

**Published:** 2022-09-24

**Authors:** Gautam Chatterji, Sourav Shukla, Suyash Singhania, Mayank P Singh, Siddhartha S Mohanty, Atin Jaiswal, Digvijay Mishra

**Affiliations:** 1 Orthopaedic Surgery, All India Institute of Medical Sciences, Bhopal, Bhopal, IND; 2 Orthopaedic Surgery, Vivekananda Polyclinic and Institute of Medical Sciences, Lucknow, IND; 3 Orthopaedic Surgery, Bhabha Atomic Research Centre and Hospital, Mumbai, IND; 4 Orthopaedic Surgery, Sir Gangaram Hospital, Delhi, IND; 5 Orthopaedic Surgery, Hi-Tech Medical College and Hospital, Bhubaneswar, IND; 6 Orthopaedic Surgery, Avadh Hospital, Lucknow, IND; 7 Orthopaedic Surgery, Ramkrishna Sevashram Hospital, Mirzapur, IND

**Keywords:** harris hip score, total hip replacement, bipolar hemiarthroplasty, elderly patients, femoral neck fracture

## Abstract

Introduction

Displaced fractures of the neck of femur in elderly patients usually require surgical intervention, with either bipolar hemiarthroplasty (BHA) or total hip replacement (THR). However, there is still controversy regarding the optimal prosthesis. The present study was performed to compare the functional outcome of BHA versus THR in elderly patients with displaced fracture of the neck of femur.

Materials and methods

This prospective study was conducted between December 2019 and December 2021. This study included 40 patients with displaced fracture of the neck of femur. All patients were more than 60 years of age. The patients were randomly allocated to be treated with either BHA or THR. Functional assessment was done using Harris hip scores at one month, three months, six months, and one year postoperatively.

Results

In our study, at all follow-ups, the Harris hip score was found to be more in patients in the THR group than in the BHA group. In the BHA group, the mean Harris hip scores were 59.95, 66.25, 68.80, and 75.70 at the follow-up visits at one month, three months, six months, and one year, respectively, while in the THR group, the mean Harris hip scores were 65.06, 69.40 72.50, and 78.19, respectively.

Conclusion

THR is a better option as compared to BHA in the management of elderly patients with fracture of the neck of femur on account of less complication rates and higher Harris hip scores.

## Introduction

In the elderly population, a fracture of the neck of femur is one of the most common fractures that results in morbidity and mortality. With increasing life expectancy worldwide, the number of elderly individuals is increasing, and it is estimated that the incidence of hip fractures will also rise and will pose an epidemic problem [1]. Fracture of the neck of femur in elderly individuals occurs most commonly after minor falls or twisting injuries [2]. Elderly individuals are more predisposed to such fractures due to osteoporosis, malnutrition, decreased physical capabilities, and multiple comorbid diseases [3-5]. There is still controversy regarding the optimal prosthesis for the management of displaced femoral neck fractures in elderly patients.

The treatment goal should be to return the patient to his/her pre-morbid status of function [6]. This can be achieved by the use of a primary prosthetic replacement with either bipolar hemiarthroplasty (BHA) or total hip replacement (THR). Therefore, the present study was performed to compare the functional outcome of BHA versus THR in elderly patients with displaced fracture of the neck of femur.

## Materials and methods

This prospective study was conducted in the Department of Orthopaedics of a tertiary care hospital between December 2019 and December 2021. Institutional ethical committee clearance was obtained prior to the conduction of the study. Patients presenting to the emergency department with displaced fractures neck of the femur were admitted to this hospital. All admitted inpatients were subjected to full evaluation which included clinical, radiology, and biochemistry, and received standard primary care.

The inclusion criteria were as follows: displaced fracture of the neck of femur, age over 60 years, independent walking prior to the injury, and no contraindication to anesthesia. The exclusion criteria were as follows: osteoarthritis or rheumatoid arthritis in the fractured hip, pathological fracture, and non-ambulatory patients prior to the injury.

A total of 40 patients were included in the study. The patients were then randomly divided into two groups of 20 patients each using random number tables generated online. Group A was treated with BHA and group B with THR. In all patients, till the day of surgery, skin traction was applied to the fractured lower limb, with the aim of decreasing pain and preventing shortening. Radiographs of the pelvis with bilateral hips, anteroposterior view, were taken for all the patients. Patients as well as the next of kin were explained about the surgery, and written informed consent for the surgery was obtained from all patients.

Patients were prepared for surgery after obtaining anesthesia fitness. Half an hour prior to skin incision, intravenous antibiotic prophylaxis with 1.5g of cefuroxime was administered to all patients. All hips were operated on with Moore's posterior approach. Prophylaxis against deep venous thrombosis was started 12 hours before surgery and continued as per standard protocol for 30 days postsurgery. Rehabilitation was similar for both groups. Within the first three days after surgery, active exercises and full weight bearing were initiated. Patients were usually discharged after five to seven days of surgery. Rehabilitation was continued during the study period. Patients were reviewed at regular intervals of one month, three months, six months, and one year. At each follow-up, Harris hip score was used to carry out the functional assessment.

Statistical analysis

The data were presented in terms of mean ± standard deviation, frequencies, and percentages. To compare categorical variables between the groups, the chi-square test was used. To compare continuous variables between the groups, the unpaired t-test was used. To compare the mean change in scores, paired t-test was used. A p-value of less than 0.05 was considered statistically significant.

## Results

There were a total of 40 patients in our study, out of which 20 patients underwent BHA and 20 patients underwent THR. The demographic attributes of all the patients are present in Table 1. In our study, all the patients in both groups were more than 60 years of age. The mean age of the patients in the BHA group was 68.50 years, and in the THR group, it was 70.38 years. In our study, females constituted 60% and males constituted 40% of total patients. The bipolar group included seven (35%) males and 13 (65%) females and the THR group included nine (45%) males and 11 (55%) females. Most of the patients had comorbidities such as diabetes mellitus, hypertension, cardiovascular problems, and pulmonary diseases. These comorbidities can have an adverse effect on the functional outcome. However, in our study, it was observed that there was no significant difference in the number of comorbidities between the two groups.

**Table 1:** Patient demographics THR, total hip replacement

	Age(years)	Sex	
		Male	Female
Bipolar	68.50 ± 7.73	7	13
THR	70.38 ± 8.09	9	11

The detailed surgery data of the patients are given in Table 2. The mean duration of surgery was significantly higher in the THR group (133.19 minutes) when compared to the BHA group (55.95 minutes). The mean blood loss was significantly higher in the THR group (569.06 ml) when compared to the BHA group (334.75 ml). The mean amount of blood transfusions was higher in the THR group (seven patients) when compared to the BHA group (four patients). The differences between the two groups in terms of duration of surgery, blood loss, and amount of blood transfusion were statistically significant (p<0.05). Thus, in relation to the duration of surgery, total blood loss during surgery, and amount of blood transfusion, BHA was found to be better than THR.

**Table 2:** Operative record of patients THR, total hip replacement

	Bipolar	THR	p-Value
Duration of surgery (minutes)	55.95 ± 7.01	133.19 ± 21.02	<0.0001
Blood loss (ml)	334.75 ± 38.91	569.06 ± 49.13	<0.0001

**Figure 1:**
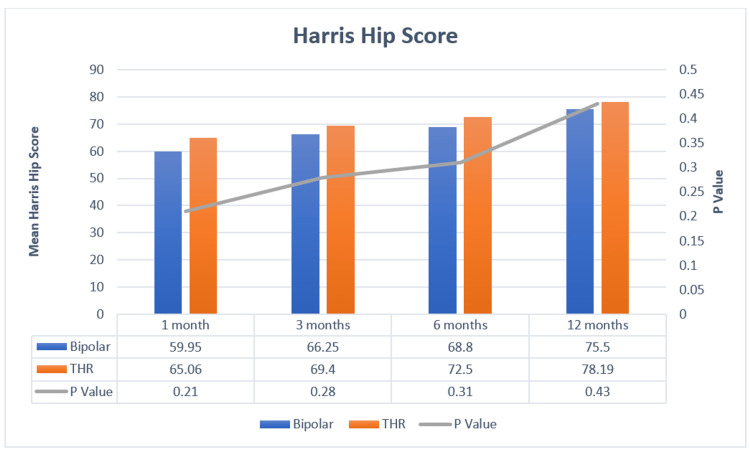
Comparison of Harris hip scores between the groups across the time periods THR, total hip replacement

The functional outcome of patients in both groups was analyzed using the Harris hip score during their follow-up at one month, three months, six months, and one year, and is represented in Figure 1. In the group that underwent BHA, the mean Harris hip score was 59.95, 66.25, 68.80, and 75.70 at the follow-up visits at one month, three months, six months, and one year, respectively. In the group that underwent THR, the mean Harris hip scores were 65.06, 69.40, 72.50, and 78.19 at the follow-up visits at one month, three months, six months, and one year, respectively. At all follow-ups, the Harris hip score was found to be more in patients of the THR group than in the BHA group.

## Discussion

The treatment of displaced fracture of the neck of femur in elderly patients is still a subject of discussion. According to previous studies, BHA is preferentially performed in older patients with limited life expectancy and low functional demands. THRs are procedures that need more precision and time. They can give better functional results and are better suited for patients who are younger, have a higher life expectancy, and have more functional demand [7,8].

In this study, we found that the mean age of patients in both the groups was similar and there was not much difference. There was no significant difference in the gender between the groups showing comparability of the groups in terms of gender; however, a female predominance was seen in both BHA (65%) and THR (55%) groups.

In this study, we found that the duration of surgery, blood loss, and amount of blood transfusion were considerably less in the BHA group. These findings corroborate with the results of multiple studies and reviews on the topic conducted in the past both in the western and Indian populations. In elderly patients, the treatment outcome is significantly affected by the duration of surgery and the amount of blood loss. Increased duration of surgery and more blood loss make these patients more susceptible to infection. However, in our study, we found that although THR was associated with increased duration of surgery and blood loss when compared to BHA, complications were reportedly higher in the BHA group, which was in agreement with observations made by Ossendorf et al. and Dawson et al. [7,9]. In our study, we observed that the total duration of hospital stay was not consistent between the two groups and, therefore, was not considered reliable. This was comparable to observations made by Wang et al. [10].

Burgers et al. published a meta-analysis and review of randomized controlled trials comparing modes of arthroplasty in fracture of the neck of femur [11]. The Harris hip score was used to compare the functional outcome. The total score was found to be significantly higher in THR as compared to other modes of arthroplasty. The range of Harris hip score is from 0 to 100 points. It includes the following subdomains: pain, deformity, function, and range of motion. These subdomains have been used in many studies all over the world to give a clear picture of the patient being assessed. In the same review, both pain and function scores were found to be better in patients who underwent THR. In our study, we found that the mean Harris hip scores at end of one month, three months, six months, and one year for THR were higher. The comparison of scores at specific time periods did not have statistical significance, but the score of patients undergoing THR was slightly higher. Scores for the subdomain of pain, function, and range of movement were higher in patients managed by THR.

Various complications have been noted in various studies describing arthroplasty to manage neck femur fractures. Infection, deep vein thrombosis, and delayed mobilization are common immediate complications. Implant failure, dislocation, periprosthetic fractures, and loosening of the implant are common late complications. Certain complications are associated specifically with the mode of arthroplasty used [11,12]. In our study, we noted down any immediate or late postoperative complications. Wound infections were the most common complication with both BHA and THR [13]. There was one case of deep wound infection in the patients managed by BHA. For this patient, thorough wound debridement was performed and appropriate intravenous antibiotic was given. After about two weeks of treatment, the wound infection completely healed. There was one case of superficial wound infection in the THR group. The patient was treated with oral antibiotics and dressings. According to a recent review on arthroplasty in fracture of the neck of femur by Khan et al., acetabular wear, protrusion of the femoral head into the acetabulum, and pain due to implant impingement are common in BHA [14]. Risk of dislocation is more common in total hip arthroplasty. In our study, no dislocation occurred in either of the two groups. Chronic hip pain was seen in three patients managed by BHA. This was probably due to cartilage damage and erosion of the acetabular surface. In the literature, bipolar arthroplasty is associated with a higher rate of revision surgery when compared to THR. This high revision rate is due to acetabular erosion [11]. However, in this study, there were no cases of revision surgery, and the reason for this is short duration of follow-up.

Limitations

The main limitations of this study were a small sample size and short follow-up period. For a proper study of postoperative complications, a longer period of observation is required.

## Conclusions

THR is a better option as compared to BHA in the management of elderly patients with fracture of the neck of femur. Hence, in elderly patients with displaced fracture of the neck of femur, a THR can be considered as primary treatment modality over BHA on account of less complication rates and higher Harris hip scores.
